# Combined Gaze Metrics as Stress-Sensitive Indicators of Microsurgical Proficiency

**DOI:** 10.1177/1553350620942980

**Published:** 2020-07-20

**Authors:** Jani Koskinen, Roman Bednarik, Hana Vrzakova, Antti-Pekka Elomaa

**Affiliations:** 1 School of Computing, 163043 University of Eastern Finland, Finland; 2 Microsurgery Center, 60650 Kuopio University Hospital, Finland; 3 Department of Neurosurgery, Institute of Clinical Medicine, 60650 Kuopio University Hospital, Finland

**Keywords:** eye tracking, microsurgery, expertise, cognitive workload, pupil dilation, blinking

## Abstract

*Background.* Evaluation of microsurgical proficiency is conventionally subjective, time consuming, and unreliable. Eye movement–based metrics have been promising not only in detection of surgical expertise but also in identifying actual cognitive stress and workload. We investigated if pupil dilations and blinks could be utilized in parallel to accurately classify microsurgical proficiency and its moderating features, especially task-related stress. *Methods.* Participants (n = 11) were divided into groups based on prior experience in microsurgery: novices (n = 6) with no experience and trained microsurgeons (n = 5). All participants conducted standardized suturing tasks with authentic instruments and a surgical microscope. A support vector machine classifier was used to classify features of microsurgical expertise based on percentage changes in pupil size. *Results.* A total of 109 successful sutures with 1090 segments were recorded. Classification of expertise from sutures achieved accuracies between 74.3% and 76.0%. Classification from individual segments based on these same features was not feasible. *Conclusions.* Combined gaze metrics are applicable for classifying surgical proficiency during a defined task. Pupil dilation is also sensitive to external stress factors; however, the usefulness of blinks is impaired by low blink rates. The results can be translated to surgical education to improve feedback and should be investigated individually in the context of actual performance and in real patient operations. Combined gaze metrics may be ultimately utilized to help microsurgeons monitor their performance and workload in real time—which may lead to prevention of errors.

## Introduction

Microsurgical techniques are prevalent in numerous surgical disciplines, such as ear–nose–throat diseases, neurosurgery, ophthalmology, oral and maxillofacial surgery, orthopedics and plastic surgery, and vascular surgery.^1^ Microsurgical procedures utilize both optical and digital microscopes to fine-tune instrument handling in confined spaces and with sensitive tissues, requiring extreme situational awareness, fluent eye–hand coordination, and uninterrupted concentration from the operator. Despite years of training, the microsurgical procedures increase cognitive workload which in turn increases the chance of surgical errors.^2^

Microsurgical expertise spans not only specialist knowledge, understanding the anatomy and treatment procedures,^3^ but also the technical skill and practice of microsurgical conduct.^4^ Such expert surgical practice relies on mentoring and feedback from more experienced surgeons.^5^ However, the evaluation of surgeons’ proficiency is subject to numerous drawbacks, with subjectivity belonging to the commonly acknowledged issues.^6,7^ To avoid cumulative errors from subjective practice, medical practitioners have adopted various objective systems such as checklists and rating scales.^8^ In addition, assessment of surgeons’ cognitive workload and proficiency during the procedures has been an ongoing area of research for years.^6,9^

Prior research has investigated various computational approaches to objectively assess surgical skills. The reported methods have mainly involved instrument movements,^10-12^ including surgically applied forces and arm kinetics.^6,13^ Some authors, such as Grober et al and Harada et al, investigated microsurgery specifically. Several studies have also reported the specific gaze patterns of training surgeons.^14-16^ In Ref. 17, the authors used several eye metrics, including the Index of Cognitive Activity pupil dilation and blink rate and managed to successfully classify surgeons as experts and nonexperts using linear discriminant analysis and nonlinear neural networks. Likewise, in Ref. 18, the authors investigated the percentage change in pupil size (PCPS) and the Index of Pupillary Activity in addition to traditional gaze metrics such as the fixation rate and found these to be capable of differentiating surgeons’ skill level during live surgery. In Ref. 19, the increased difficulty in a laparoscopic task was found to correlate with peak pupil dilation in a group of novice participants. In Ref. 20, the authors used pupil size as a metric for mental workload when comparing bimanual and unimanual performance during a simulated endoscopic task.

The use of pupil dilations for assessing expertise and workload is justified by the phenomenon of *task-evoked pupillary response*, where an increased processing load causes the pupil to dilate.^21^ The task-evoked pupillary response has been validated in many different contexts involving attention, memory, and perception.^22^ Similarly, the increased mental workload has been found to correlate with changes in blinking patterns.^23,24^ Higher mental workload and stress have been reported to correlate with experience in driving,^25^ in simulated aviation tasks,^26^ and during surgery.^27^ Consequently, the differences in workload experienced by novice and expert surgeons should lead to differences in pupil dilations and blink rates.

With a custom eye tracker embedded into a surgical microscope, we recorded the blinks and pupil dilations of novice and expert microsurgeons as they performed a set of microsurgical training sutures. In our previous research,^28,29^ we found that novices estimated the suturing task to be significantly more demanding than experts and that there are differences in pupil dilations and blink rates between these 2 groups. Here, we extend this research by studying the combined applicability of pupil dilation and blink rate to classify expertise at suture- and segment-level features. Our hypothesis is that the blink rate and pupil dilation are best used in parallel to account for both proficiency and cognitive workload during microsurgery.

## Materials and Methods

### Participants and Cognitive Workload Evaluation

We recruited a total of 11 participants for the study (Table 1). All participants (2 females and 9 males; mean age = 30.91 years and SD = 6.19) had normal or corrected-to-normal vision. We divided the participants into novices and experts based on their previous experience in microsurgery. The novices had no microsurgical experience, whereas the experts were plastic surgeons performing 30-60 surgical operations a month using a surgical microscope or loupes. Some novices had medical training and some surgical experience outside of microsurgery. One novice reported high surgical expertise, resulting in a high standard deviation in the novice group. The experts were recruited from a plastic surgery clinic, and the novices were staff at the surgical simulation laboratory where the experiment took place.

**Table 1.** Overview of the 2 Groups. Surgical and Microsurgical Skills Are Reported in Number of Months, Averaged over the Participants. One Novice Reported High Surgical Expertise, Resulting in High SD of the Novice Group. SURG-TLX Scores Are Based on Self-Reports from the Participants. Standard Deviations Are Given in Parenthesis.

	Group	Novice	Expert
Demographic	Gender	2 females and 4 males	5 males
	Age (year)	30.50 (8.26)	31.40 (1.36)
	Surgical practice (month)	60.00 (91.65)	61.20 (19.03)
	Microsurgical practice (month)	.00 (.00)	31.20 (24.71)
SURG-TLX	Mental demands	13.06 (2.17)	8.34 (3.91)
	Physical demands	14.88 (2.61)	5.82 (2.51)
	Temporal demands	8.70 (4.54)	7.76 (1.55)
	Task complexity	12.50 (6.09)	5.44 (2.28)
	Situational stress	14.30 (1.03)	8.50 (4.65)
	Distractions	2.50 (2.77)	6.14 (3.50)
	Sum	65.94 (10.48)	42.00 (12.32)

Abbreviation: SURG-TLX = surgery task load index; SD = standard deviation.

At the start of the experiment, the participants received instructions and signed a consent form before starting the task. We also took measures to eliminate potential external factors that could affect the pupil dilations and the blink rate. We asked the participants to refrain from using stimulants such as coffee before the experiment. During the instruction phase, the participants rested at least 10 minutes before the eye tracker calibration. Each participant adjusted the ergonomics of the seat and the microscope to their personal preferences. Throughout the experiment, the illumination of the microscope and the room was kept even.

After the experiment, the participants filled the surgery task load index (SURG-TLX) instrument.^30^ The SURG-TLX instrument uses a 20-point Likert scale to measure surgical workload in 6 dimensions: mental, physical, and task demands, as well as task complexity, situational stress, and distractions. Results for the novices and experts are described in Table 1.

### Suturing Task

The training task board was designed iteratively by experienced surgeons in collaboration between 2 intercontinental university hospitals. The task cardboard had 2 rows with 3 slots each, in total 6 slightly different suturing subtasks. Each column has predefined direction (0 – 45 – 90–degree angles), and the second row repeats them under higher magnification. To each incision, the participants completed 2 sutures. The card is designed to intuitively force the performer to adjust for different magnifications and field of view, which are typically encountered in microsurgical suturing.

**Figure 1.**
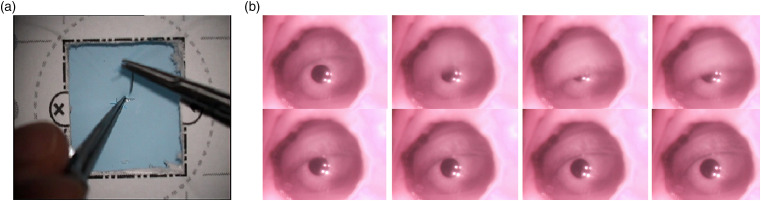
(A) Scene under the microscope and (B) the eye during a blink.

The participants conducted the sutures using high-quality microsurgical needle holders and suturing forceps with 9.3 mm 3/8 taper head needles attached to 7-0, 50-cm polypropylene monofilament sutures. The participants used a Zeiss OPMI Vario S88 surgical microscope with an embedded custom-made eye tracker. The eye tracker had a sampling rate of 30 Hz and was installed on the right ocular of the microscope. Figure 1 shows the scene under the microscope and the view from the eye tracker.

### Data Processing and Segmentation

Each suture was divided into segments defined by an expert microsurgeon (Table 2). The segment names indicate the event that marked the start of a new segment. For example, “needle pick” begins when the needle is picked with the needle holder and ends when the needle touches the edge of the cut, which marks the start of “edge touch.”

**Table 2.** Description of the Suture Segmentation. Except for the Cutting Segment, the Event Marking the Start of the Segment Is Given in the Description, and the End of the Segment Is Marked by the Next Event in the List. The Cutting Segment Starts after Knot 3 and Ends when both Ends of the Thread Are Cut.

Suture Segment	Description
Needle pick	A needle is picked up with a needle holder or a loaded needle holder is brought to the field of view
Edge touch	An instrument or the needle touches the edge of the target surface
Pierce	The needle tip pierces the first surface wall
Needle push and pull	The needle tip penetrates the second surface wall
Extraction	A needle holder grabs the needle on the edge of the needle
Thread handling	The base of the needle penetrates the second wall
Knot 1-3	A nondominant instrument grabs the thread for suturing (this hand can pull thread also after this time point)
Cutting	Both suture threads are cut

The pupils were detected with a custom Hough transform–based algorithm, as described in Ref. 28. Traditional blink detection methods did not deliver satisfying results due to the custom-made setup of the eye tracker, and thus, we opted to filtering the blinks out manually. The participants regularly moved away from the microscope to pick the scissors at the end of the suture, and these frames were excluded when calculating the blink rate. Postprocessing of both the pupil and blink data was implemented in Python using Pandas^31^ and NumPy^32^ libraries.

We applied linear interpolation to ensure that each participant had a constant sampling rate of 30 Hz. After resampling the data, we applied a low-pass filter at a 4-Hz cutoff frequency, as frequencies above 2 Hz can be considered noise.^33^ Movement of the hands and the tools under the microscope could also affect the amount of light coming to the eye. We estimated the illumination levels from grayscale frames extracted from the scene videos and calculated their Pearson correlation with the pupil size. The correlation on the detrended data was found to be low (|ρ| < .1).

For normalizing the pupil data, the pupil measurements are usually subtracted by or divided with a suitably chosen baseline.^34^ Here, the changes in the pupil size were calculated as a PCPS compared to the baseline, as defined in^35^

(1) $$\text{PCPS}=\;\frac{X-\mu }{\mu }$$

where *X* is the pupil size and *μ* is the baseline pupil size. A new baseline was calculated for each of the 6 slots in the training board, from a 200-frame window before the first suture to that slot was started. Calculating the baseline before each suture was not possible because participants often started the second suture right after the first one within a slot.

### Machine Learning for Automatic Classification of Expertise

Ideally, a system that uses eye tracking data to evaluate microsurgical expertise should work in near real time. To this end, we studied the performance of classifiers that use simple features extracted from pupil dilation data. The features chosen were the average percentage change in pupil size (APCPS) and the standard deviation of the percentage change in pupil size (SDPCPS) in each segment and the blink rate in blinks per minute.

The classifier performance was evaluated at segment and suture levels. At the segment level, the classifier was given features from individual segments, and at the suture level, the classifier was given features from all the segments that make up that suture. We first pilot tested various classifiers (logistic regression, linear discriminant analysis, k-nearest neighbors, decision tree, Gaussian Naive Bayes, support vector machines (SVMs), AdaBoost, and Gaussian process) with default parameters to estimate classifier performance. Considering the sample size, distributions of the feature variables, and between-participant differences, we chose to do the classification using the SVM classifier.

Before training the classifier, the training and test sets were scaled separately to have a zero mean and unit variance. To find an optimal value for the penalty parameter C that determines the cost of misclassifications, we followed the guidelines given in Ref. 36 and ran exponential grid-search k-fold cross-validation with the parameter range [2^−3^, 2^−2^, …, 2^17^] and fold size k = 10. The parameter search was done on both segment- and suture-level classification schemes separately, after which we chose one value that was used for all classification. The optimal penalty parameter C was found to be .25.

**Figure 2.**
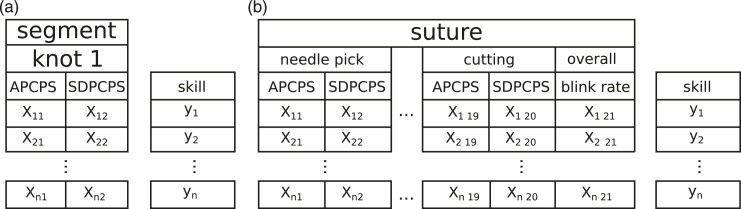
Scheme for classifying expertise from segments and sutures. For segments (A), we train and test the classifiers for each segment individually (here, knot 1). In the suture classification (B), data from all the segments are used.

In the segment-level classification, each segment was evaluated individually with APCPS and SDPCPS as features. In other words, we assume that the segment from which the feature values come from is known. The observed blink rate was too low to make it useful in classifying individual segments. For suture-level classification, the features were APCPS and SDPCPS from each of the 10 segments that make up the suture and the blink rate for the complete suture, with a total of 21 features and 109 sutures. Since the pupil features are likely to be correlated in nearby segments and because the large number of pupil features would diminish the applicability of the blink rate, we also tested dimensionality reduction using principal component analysis (PCA). Figure 2 shows an overview of the classification scheme.

At both classification levels, we used repeated stratified k-fold cross-validation with a fold size of 10 and 10 repetitions to evaluate classifier performance. We also tested the effects of individual participants by repeating the suture-level classification and each time leaving out one participant. The parameters used to evaluate classifier performance are as follows

1. Accuracy: Percentage of participants classified correctly as a novice or an expert;
2. True positive rate: Percentage of experts correctly classified as experts;
3. False positive rate: Percentage of novices classified as experts; and
4. Precision: Percentage of true experts out of all participants classified as experts.

In all, 55% of the data belonged to experts, which can be taken as the baseline accuracy for classification. Python package Scikit-learn was used to perform the training of the statistical models and classification.^37^

## Results

Data from one of the novice participants were discarded because of technical issues, leaving 5 novice and 5 expert participants. Two novice participants failed to complete all the sutures, and the unsuccessful sutures—11 in total—were left out of the final analysis. Thus, 109 sutures were completed successfully, of these, 60 by expert participants. As each suture consisted of 10 segments, there were a total of 1090 segments. Five segments had missing data that we replaced with the mean value of similar segments from the same participant.

Mean duration of a suture for experts was 70.6 seconds (SD = 14.9) and for novices, 168.8 seconds (SD = 68.7). According to a two-tailed t-test, this difference is statistically significant, t(108) = 10.78, *P* < .001. The mean blink rate per suture was 4.69 blinks/min (SD = 5.04) for experts and 4.68 blinks/min (SD = 3.62) for novices.

### Assessing Expertise from Segments

Results of segment-based classification are displayed in Table 3. Considering the baseline accuracy of 55%, it is evident that segment-based classification using these features does not perform sufficiently well. The recall and precision values show that most participants were classified as experts. Best accuracies were achieved at the beginning and toward the end of the suture, but even then, the performance is only slightly above the baseline.

**Table 3.** Results for Segment-Based Classification of Expertise Using Support Vector Machines with Average Percentage Change in Pupil Size and Standard Deviation of the Percentage Change in Pupil Size as Features in Each Segment.

Segment	Accuracy	Acc. 95% CI Low	Acc. 95% CI High	TPR	FPR	Precision
Needle pick	.555	.545	.565	.968	.953	.556
Edge touch	.663	.640	.686	.883	.606	.647
Pierce	.540	.530	.550	.962	.975	.547
Needle push	.551	.548	.554	1.000	1.000	.551
Extraction	.551	.548	.554	1.000	1.000	.551
Thread handling	.539	.531	.547	.975	.996	.545
Knot 1	.614	.594	.635	.882	.713	.606
Knot 2	.539	.528	.549	.952	.968	.546
Knot 3	.597	.578	.617	.922	.802	.588
Cut	.624	.602	.645	.908	.725	.609

Abbreviation: CI = confidence interval.

### Assessing Expertise from Sutures

The performance of suture-level classification is given in Table 4. The table also provides results of classification with only the pupil features and with the dimensionality of the pupil features reduced. PCA was applied to the APCPS and SDPCPS features separately. With 4 principal components for APCPS and SDPCPS each, the classification results were on par with the results from using the entire set of features. However, inclusion of the blink rate as one of the features did not significantly improve the results. Nevertheless, the achieved accuracies of 74.3%-76.0% are promising, and together with recall and precision values, they show an apparent improvement over segment-based classification. In addition, the trained classifier generalized well with minor variance. As can be seen in Table 5, the achieved classification rates varied only modestly in the leave-one-participant-out cross-validation.

**Table 4.** Results for Suture-Based Classification of Expertise Using Support Vector Machines with Average Percentage Change in Pupil Size, Standard Deviation of the Percentage Change in Pupil Size for Each Segment (10 + 10 Features Total), and the Blink Rate for the Entire Suture as Features. Principal Component Analysis Was Applied to Reduce the Number of Pupil Features.

Accuracy	Acc. 95% CI	Precision	TPR	FPR	Feature Set	Number of Features
.767	.744-.789	.771	.858	.347	APCPS and SDPCPS	10 + 10
.747	.723-.770	.740	.843	.3715	PCA (APCPS, 4 principal components), PCA (SDPCPS, 4 principal components)	4 + 4
.744	.719-.769	.747	.833	.365	APCPS, SDPCPS, and blink rate	10 + 10 + 1
.738	.715-.762	.745	.830	.373	PCA (APCPS, 4 principal components), PCA (SDPCPS, 4 principal components), and blink rate	4 + 4 + 1

Abbreviation: APCPS = average percentage change in pupil size; SDPCPS = standard deviation of the percentage change in pupil size; PCA = principal component analysis; CI = confidence interval TPR = true positive rate, FPR = false positive rate.

**Table 5.** Classification from Sutures with One Participant Left Out, with APCPS, SDPCPS for Each Segment and Blink Rate for the Entire Suture as Features.

	Participant Excluded	Accuracy	Acc. 95% CI Low	Acc. 95% CI High	TPR	FPR	Precision	Sample Size
1	Expert	.723	.695	.750	.683	.237	.769	97
2	Expert	.720	.693	.747	.816	.373	.694	97
3	Expert	.792	.767	.816	.819	.235	.794	97
4	Expert	.740	.716	.764	.725	.246	.769	97
5	Expert	.761	.732	.791	.773	.250	.763	97
6	Novice	.759	.734	.784	.857	.379	.773	103
7	Novice	.767	.742	.792	.878	.414	.790	97
8	Novice	.777	.755	.800	.907	.432	.786	97
9	Novice	.773	.748	.797	.905	.416	.769	102
10	Novice	.771	.749	.793	.883	.412	.794	97

Abbreviation: CI = confidence interval TPR = true positive rate; FPR = false positive rate.

## Discussion

Eye metrics present a new platform for monitoring microsurgeon’s cognitive workload and performance. In this work, we specifically investigated the extent of combined pupil- and blink-based measures for indicating of microsurgical proficiency. We utilized a custom eye tracker, which allowed recording with unpreceded accuracy, and without limiting natural microsurgical ergonomics. Our results suggest that pupil- and blink-based metrics can support objective assessment of microsurgical proficiency, with pupil dilations being the predominant indicator of the participants’ expertise.

In the set of machine-learning experiments, we evaluated how well the SVM classifier recognizes participants’ expertise at the higher suture level and then at the finer level of suture segments. The classification of expertise based on pupil dilations at the suture level revealed greater potential. The SVM was able to classify expertise at considerably high accuracy, considering the simplicity of the features and the fact that we were only utilizing eye tracking data. Adding the blink rate as a feature did not significantly improve the classification results, most likely due to the low blink rate that was observed. The low blink rates were in line with the extremely low blink rates during microsurgery which have previously been reported.^38^ In Ref. 17, the authors used the blink rate as one of the features to successfully classify expertise in a laparoscopic task, but it is unclear how much excluding the blink rate would have affected their results.

The same classification approach at the finer level of suture segments proved to be mainly unfeasible due to the large variance that was observed even within individual participants. The highest performance was seen with segments toward the end of the suture, suggesting that this phase could potentially signal differences in expertise. For a reliable classification from suture segments, we would have to use more refined features, which on the other hand, would require a careful control of the noise.

The pupil changes were therefore indicative of microsurgical suturing proficiency when they were assessed from longer periods of time, over the entirety of the suture. With longer time, more information is used to make the classification and the process is less susceptible to noise since the effects on individual segments cancel out.^39^ Uninformative noise may occur because the pupil accommodates to changes in illumination. However, the pupil size is also affected by mental effort and motor demands, and changes in the pupil size are linked to arousal and fatigue,^22^ and especially cognitive workload.^21,40,41^ The link between pupillary responses and cognitive workload has been investigated in several studies.^34^ In the field of laparoscopic surgery, Jiang et al found that the pupil dilations corresponded to the precision of hand movements required by the task, and the increased peak and duration of the pupil dilation were associated with elevated task difficulty.^39,42,43^

One limitation of this study is the large between-participant variation, which prohibits the use of more sensitive features for classification. The large variance between individual participants also indicates the need for different types of data to create additional constraints, either more refined features from pupil data or additional data from other sources. The pupil size is also sensitive to many factors other than the cognitive workload, and future implementations of pupil-based metrics need to control these external factors. These include controllable factors such as fatigue, caffeine intake,^44^ and illumination changes but also factors affecting the mental workload and stress that are unrelated to the surgical task. To further validate the method, the results could be compared to other performance metrics. For example, expert surgeon’s poor performance could indicate an increase in cognitive workload, which might be detected as changes in the pupil size.

Regarding the segmentation scheme we used, some of the segments were extremely short, and the latencies associated with physiological measures could lead to observing the effects of increased cognitive workload in a different segment. The straightforward approach used here considered the segments as being independent of each other. There could be interrelationships between different parts of the suture that can reveal differences in expertise, and an approach that better considers the sequential nature of the data could improve the classification. Another approach would be to analyze the pupil behavior around short independent events, for example, when the participant pierces the skin with the needle.

Nevertheless, microsurgery offers an ideal platform for realizing pupil- and other eye-based metrics as more objective approaches to evaluating workload and expertise since eye tracking can be naturally integrated to the surgical workflow without a need for external detectors that could disturb the surgical performance. This also means that the same experiment could possibly be replicated in a real surgical operation. While the real surgical operation could present challenges that do not occur during a training task, the microscope also allows more control over some of the recording noise that can affect the results. Furthermore, the microscope camera enables video-based detection of hand and tool kinematics, and the eye-based metrics can be used to supplement this information—again, without adding anything new to the surgical procedure itself.

## Conclusion

Eye metrics are applicable for classifying surgical proficiency during a training task. Pupil dilation is also sensitive to external stress factors; however, the usefulness of blinks may be impaired by low blink rates. The results can be translated to surgical education to improve feedback, and the method should be investigated during real patient operations.

Our long-term goal for this research was to develop objective assessment methods, of proficiency and workload, that could be used in real time during microsurgery. These intelligent systems could be applied in future surgical systems to assist operators in achieving and keeping up an optimal workflow. Based on our results, eye tracking has potential in monitoring proficiency and surgical workload and could be already used in surgical training for augmenting feedback. Besides improving microsurgical training, our research has potential applications in the development of computationally enhanced systems for evaluating the surgeons’ workload in real time during surgical procedures. Therefore, eye metrics can be ultimately utilized to help microsurgeons monitor their performance and workload in real time—which may lead to prevention of errors.
